# Niche Overlap in Forest Tree Species Precludes a Positive Diversity–Productivity Relationship

**DOI:** 10.3390/plants14152271

**Published:** 2025-07-23

**Authors:** Kliffi M. S. Blackstone, Gordon G. McNickle, Morgan V. Ritzi, Taylor M. Nelson, Brady S. Hardiman, Madeline S. Montague, Douglass F. Jacobs, John J. Couture

**Affiliations:** 1 Department of Biology, Purdue University, West Lafayette, IN 47907, USA; kliffi.blackstone@gmail.com (K.M.S.B.); gg.mcnickle@gmail.com (G.G.M.); mritzi@purdue.edu (M.V.R.); 2 Center for Plant Biology, Purdue University, West Lafayette, IN 47907, USA; 3 Department of Entomology, Purdue University, West Lafayette, IN 47907, USA; taylorm@purdue.edu; 4 Department of Forestry and Natural Resources, Purdue University, West Lafayette, IN 47907, USA; hardimanb@purdue.edu (B.S.H.); madeline.montague@gmail.com (M.S.M.); djacobs@purdue.edu (D.F.J.); 5 Environmental and Ecological Engineering, Purdue University, West Lafayette, IN 47907, USA

**Keywords:** diversity productivity relationships, functional traits, hardwood forests

## Abstract

Niche complementarity is suggested to be a main driver of productivity overyielding in diverse environments due to enhanced resource use efficiency and reduced competition. Here, we combined multiple different approaches to demonstrate that niche overlap is the most likely cause to explain a lack of overyielding of three tree species when grown in different species combinations. First, in an experimental planting we found no relationship between productivity and species diversity for leaf, wood, or root production (no slope was significantly different from zero), suggesting a lack of niche differences among species. Second, data extracted from the United States Department of Agriculture Forest Inventory and Analysis revealed that the species do not significantly co-occur in natural stands (*p* = 0.4065) as would be expected if coexistence was common across their entire range. Third, we compared trait differences among our species and found that they are not significantly different in multi-dimensional trait space (*p* = 0.1724). By combining multiple analytical approaches, we provide evidence of potential niche overlap that precludes coexistence and a positive diversity–productivity relationship between these three tree species.

## 1. Introduction

Species coexistence has a well-developed body of theory [1,2,3], but is challenging to study empirically since it cannot be observed directly [4,5,6]. This challenge is particularly true for long-lived species such as tree species where processes related to coexistence or exclusion take place over long time scales. To overcome this limitation, theories are frequently employed to identify experimental results that are indirectly indicative of coexistence [7,8,9]. While this approach cannot capture all drivers of coexistence, especially mechanistic ones, it helps generate hypotheses for further testing. Coexistence is thought to depend on differences among species across various dimensions—such as resource use, habitat, seasonality, and interactions—collectively known as the niche [2,10]. Because niches are multi-dimensional and hard to measure directly, indirect indicators of niche differences or similarities are often used. While these methods cannot independently confirm or refute coexistence, they provide valuable evidence to guide future research. While there are multiple approaches to infer coexistence, they are rarely combined in one study, and an important question remains as to whether different approaches to determine coexistence agree on the same outcome [2,11]. If there is agreement between different approaches, then significant progress has been made towards a general predictive theory of community ecology.

One experimental approach to infer niche partitioning is to examine the relationship between species richness and community productivity, or the diversity–productivity (DP) relationship [12,13]. This approach focuses on community-level patterns of diversity and productivity. While not universal, researchers have found that more species-rich plant communities are also more productive [9,14]. The coexistence-related explanation for a positive DP relationship is that competition within a species is more intense than competition among species [15,16,17,18]. Under this hypothesis, low-diversity communities are hypothesized to grow poorly due to strong negative feedback from intraspecific competition (i.e., negative DP), while increasingly species-rich mixtures grow better because of diluted negative effects of intraspecific competition caused by niche complementarity (i.e., positive DP). Since a positive DP relationship has been taken as evidence of niche partitioning, applying the same logic means that a neutral or negative DP relationship must be evidence of niche overlap among diverse species mixtures [9]. This interpretation is because a neutral DP relationship means that intra- and interspecific competition are of equal strength, and a negative DP relationship means that interspecific competition is equal or more intense than intraspecific competition. Studying non-positive DP relationships could be argued as equally, if not more, important than studying positive ones, since non-positive DP relationships seem rare [9]. Detailed study of systems with non-positive DP relationships are therefore needed to investigate the niche overlap-related hypotheses that are the opposites of the niche complementarity related hypotheses.

Alternatively, two randomly combined species may or may not have different niches, but if they never encounter each other in nature, then studying niche differences has little ecological meaning. In isolation, patterns of co-occurrence reveal little about coexistence and aggregated co-occurrence in natural ecosystems is necessary, but not a sufficient condition, to infer coexistence. But when observational data about co-occurrence are combined with evidence from other approaches, we suggest the combination provides an even richer insight into natural patterns of coexistence. One such method is the checkerboard score (C-score), named after the appearance of a perfect negative occurrence matrix (e.g., $\left[\begin{array}{cc} 1 & 0\\ 0 & 1 \end{array}\right]$) [19,20]. By comparing the observed C-score to a null distribution, one can determine whether species are (i) aggregated, as might be expected if they have niche differences and coexist in similar habitats; (ii) segregated, as might be expected if they have overlapping niches and do not naturally coexist; or (iii) if they are randomly distributed, as might be the case when nature is out of equilibrium, or species distributions are governed by neutral processes.

Lastly, functional traits have been hypothesized to serve as a surrogate for estimating the n-dimensional hypervolume that defines the niche space of a species. The logic is that if natural selection has shaped the form and function of species traits, then differences in trait space might reflect differences in niche space [8]. Thus, species that coexist are hypothesized to have significantly different functional trait space, while those that coexist would have functional trait space that are not significantly different.

Here, we combine these three approaches to identify niche overlap in three North American hardwood tree species: American chestnut (*Castanea dentata*), black cherry (*Prunus serotina*), and northern red oak (*Quercus rubra*). First, we will determine the DP relationship in a common garden, mixed-species planting after over a decade of growth. We hypothesize that a positive relationship would exist and be indicative of niche partitioning, and also hypothesize that any form of non-positive relationship would be indicative of niche overlap. Second, we will examine the co-occurrence patterns of the three species across their entire native range with the United States Department of Agriculture (USDA) Forest Inventory and Analysis (FIA) data. We hypothesize that patterns of co-occurrence in nature should also align with conclusions about niche partitioning and coexistence from the DP relationship. Finally, we will examine how these species differ in six globally important functional traits to gain insight into how trait space differences or similarities influence coexistence or exclusion. We will combine these outcomes with a detailed review of the natural history and morphology of our study species (Appendix A). Ultimately, we will assess if these three approaches can be combined in a complimentary approach to determine the probability of coexistence for three hardwood tree species.

## 2. Results

### 2.1. Diversity–Productivity Relationship

**Figure 1:**
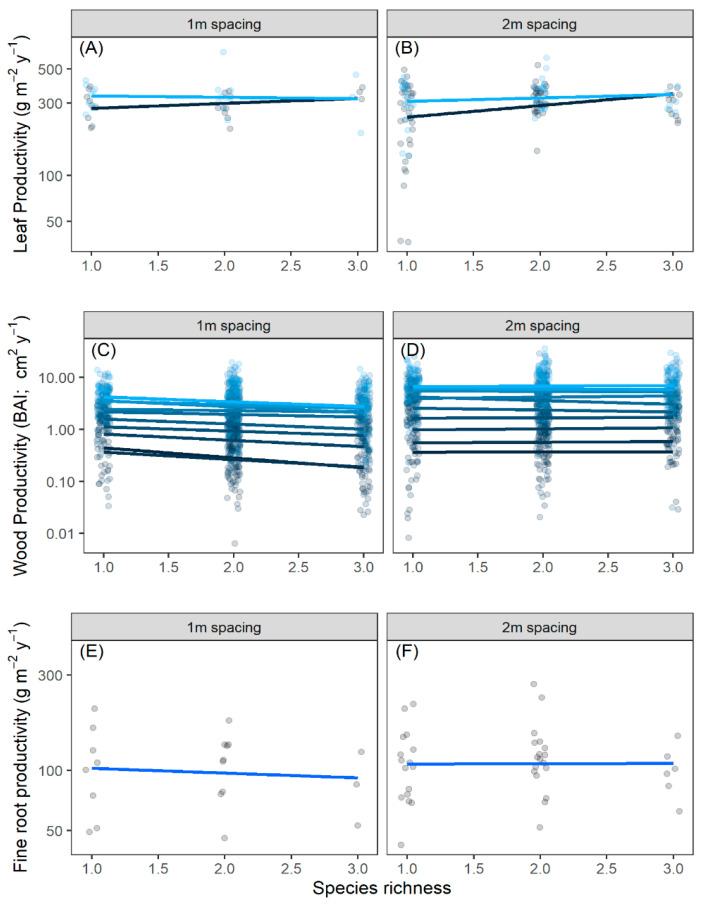
The relationship between species richness and wood productivity estimated from leaf litter traps from 2018 to 2019 (**A**,**B**), dendrochronology as basal area increments from 2007 to 2017 (BAI; (**C**,**D**)), and fine root productivity in 2019 (**E**,**F**). The *y*-axis is on a log_10_ scale in all panels. Trees were spaced either 1 m or 2 m apart (1 tree/m^2^, or 0.5 trees/m^2^, respectively). Points are drawn with a jitter around richness, and the *y*-axis is plotted on a log_10_ scale. Lines come from a linear regression (Supporting Information Tables S1–S3). None of the estimates for the individual slopes of the diversity–productivity relationship were different from zero. Different colored symbols represent different years.

Leaf production was not correlated with diversity, density, year, or their interactions for leaf litter in 2018 and 2019 in the linear mixed effects model (Table 1; Figure 1A,B), indicating a lack of any diversity–productivity relationship (Supporting Information Table S1). The lack of difference between years as a main effect indicated that trees produced similar amounts of leaves in both years. The diversity–productivity relationship for wood production (estimated as BAI) demonstrated no significant trend in all 10 years for which we had data (Figure 1C,D; Supporting Information Table S2). Wood production differed significantly across years as a main effect ($F_{10,1733}=35.4,p<0.0001$), but this was the only significant effect (Table 1, Figure 1C,D; Supporting Information Table S2) in the model. The significant year effect simply reflects that the trees grew larger from the period when they were planted as bare-root seedlings to when the forest matured into a closed canopy forest. Fine root productivity in 2019 had no relationship with diversity or density (Table 1; Figure 1E,F). As with leaves and wood, the diversity–productivity relationship for fine roots had a slope that was not statistically different from zero (Supporting Information Table S3). The coefficients of all linear models for all tissues (i.e., for the actual slope of each line shown in Figure 1) are shown in the Supplementary Materials (Supporting Information Tables S1–S3).

### 2.2. Patterns of Co-Occurrence

**Figure 2:**
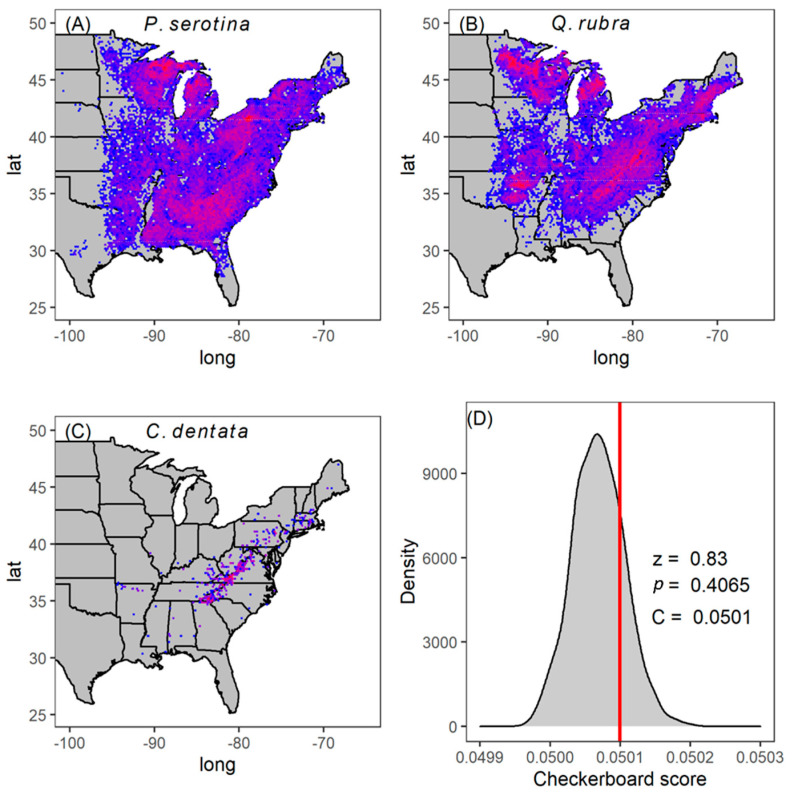
Distribution and population density (count) in natural forest stands of *P. serotina* (**A**) *Q. rubra* (**B**) and *C. dentata* (**C**) across the FIA plots in the USA. Red represents higher density, while blue represents low density. We compared observed C-score (red vertical line) with the null expectation of random distribution (grey histogram) across all FIA plots (**D**). The C score is high (relative to null) when species negatively co-occur, and low (relative to null) when they positively co-occur. The lack of difference compared to null (*p* = 0.4065) suggests these three species are randomly distributed in nature with respect to each other on average. The narrow range on the *x*-axis is due to the enormous number of plots in the FIA data.

A total of 166,119 individual trees of our three focal species were present in 7046 plots across the eastern USA (Figure 2A–C). We found that, at the scale of a 670 m^2^ FAI plot, the observed co-occurrence pattern was not significantly different from random (Figure 2D; $C_{n}=0.0501$, z=0.83, p=0.4065). This outcome means that, on average, these species do not positively co-occur throughout their range as might be expected if coexistence was common due either to niche partitioning or habitat filtering.

### 2.3. Functional Traits and Site Nutrient Availability

**Figure 3:**
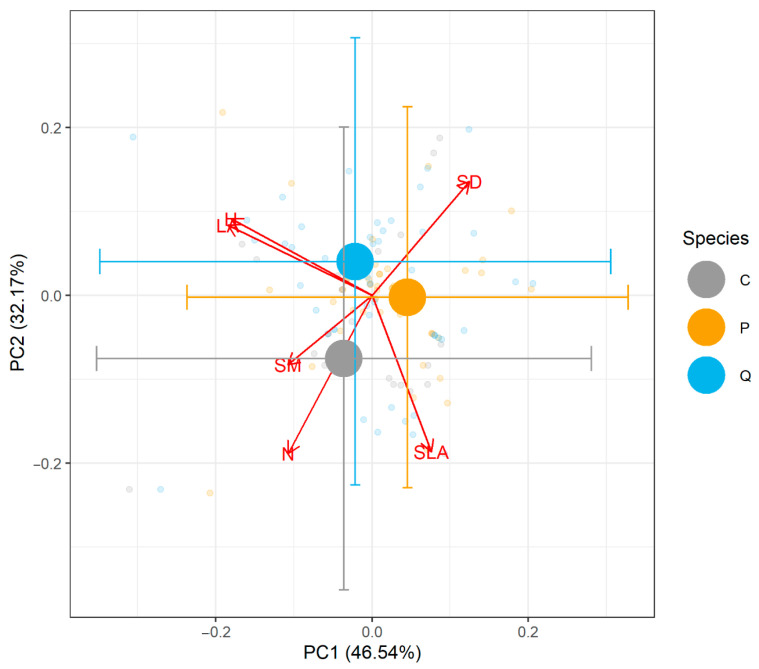
Principal component analysis showing trait data of *P. serotina*, *Q. rubra*, and *C. dentata* from TRY and measured in our plots. Large circles with error bars show the centroid of each species and 1 standard deviation for both axes. Small circles show individual observations. There were no significant differences in trait values either in ordination scores, or in raw trait values analyzed by PERMANOVA (Supporting Information Table S3, Figure S2). Species names were abbreviated to the first letter of their genus. Traits were total canopy leaf area (LA), stem density (SD), seed mass (SM), height (H), specific leaf area (SLA), and nitrogen content per dry mass of leaf (N). Missing trait values were imputed as described in the methods. A detailed review of the morphology and natural history traits of these species is also given in the Supplementary Materials.

Across the six traits included, the first two axes explained 46.7% and 35.2% of the variation in the trait data. Visualization of the PCA showed that the species had considerable similarity in multivariate trait space (Figure 3, Supporting Information Figure S2). We performed ANOVA on the scores of PC1 and PC2 and found no differences among species (Supporting Information Table S4, Figure S2). Similarly, PERMANOVA on the six-dimensional raw trait space found no significant differences among species (Supporting Information Table S4, Figure S2). A thorough review of the natural history and morphology of these species also supports the idea that *C. dentata* is a more prominent tree species based on its size, growth rate, and successional status relative to the other two species (Appendix A). Moreover, we found no influence of diversity or planting density on soil macro- and micronutrients and pollutants within this site (Supporting Information Table S5).

## 3. Discussion

In this study, we built a case that three species of hardwood trees do not appear to coexist because of niche similarity by combining multiple approaches, none of which are entirely conclusive on their own. First, the lack of a positive relationship between productivity and species richness suggests a lack of niche differences among species (Figure 1), but on its own could be explained by something other than coexistence [21]. Second, we considered the co-occurrence patterns of these species across their entire native range to ask how they are naturally distributed. This extended our analysis to the natural distribution of 166,119 individual trees across 7046 plots (i.e., 472 ha in total) spread throughout most of the range of these tree species. We found that the three study species randomly co-occur at the scale of a 670 m^2^ FIA plot other across their entire range (Figure 2). If coexistence were common due to habitat filtering or niche differences, we would have expected to find these three species aggregated more often than by chance. Finally, we were able to use six key plant functional traits to ask how these species differed from each other but found that they were functionally identical no matter how we analyzed the trait differences (Figure 3, Table S4).

Predicting species coexistence has long been a goal of ecological theory, and the confluence of multiple threads of evidence all pointing in the same direction, and consistent with multiple theoretical frameworks related to species coexistence, demonstrates that ecologists have made significant progress in uniting theory and observation surrounding species coexistence. Indeed, it was not a given that these three methods would agree to build a case about species coexistence. A failure for different approaches to agree could have suggested that ecological coexistence theory contained contradictions, and that research would be required to resolve these differences.

This study presents an unusual yet valuable finding: our most intriguing results were not statistically significant (Figure 1, Figure 2 and Figure 3). In ecological research, statistical significance at the α = 0.05 level is often viewed as a threshold for scientific discovery and publication [22,23,24]. However, in this case, the lack of significance offers critical insights into species coexistence, suggesting niche overlap, and reframes outcomes from previous studies. If niche differences are essential for coexistence, there are only two theoretical possibilities: niches are either similar or different. Both outcomes—failing to reject the null hypothesis (niches are similar, *p* > 0.05) or rejecting it (niches are different, *p* < 0.05)—carry meaningful ecological implications. Dismissing results solely because they are not statistically significant would imply that niche similarity lacks theoretical relevance, which is clearly not the case. Similarly, most previously reported diversity–productivity relationships show positive slopes [9,13,14], interpreted as evidence of niche complementarity. However, if we only recognize significant positive relationships, we overlook the equally important insights of cases where species do not exhibit niche complementarity. Additionally, when considering statistically significant outcomes, the effect size should also be considered, as statistical significance should not override biological significance. While our species pool was small, consisting of only three tree species, and may lack the power for significant statistical outcomes, recognizing these non-significant results is essential for understanding not just how species coexist, but a framework to explore why a lack of coexistence occurs.

The niche overlap we suggest for these three species matches previous work on the functional ecology of these species, which showed that these species have very similar growth rates, total leaf area, height, photosynthetic rates, and leaf nitrogen content [25], traits that are often thought to be key components of plant niches [26,27]. Although the historical range of these species has considerable overlap (Appendix A), the relative functional similarity of these species adds further support to the conclusion that they should not coexist due to niche similarity (Figure 3). Interestingly, American chestnut once was a prominent tree species in our study region, and historically made up as much as 40–50% of forest canopies [28]. However, it has been almost completely extirpated from most of its range by an invasive fungal pathogen [29]. Indeed, only 736 individual trees were present in the FIA dataset. Given that we know American chestnut was once a prominent tree species, and we know the historical ranges overlapped considerably, it stands to reason that American chestnut was competitive with the other tree species we use in this study. We argue that independently recovering this historical observation gives more strength to our conclusions about niche overlap that suggests considerable fitness differences, and to the ecological theories associated with these concepts.

An important caveat of this work is that the results from each approach we employed (i.e., the absence of a positive DP relationship, the random distribution, and lack of functional trait differences) do not independently infer niche overlap of these three species. Multiple factors can influence each of these approaches, including the spatial scale of measurements, environmental variation, stand ontogeny, biotic filtering, and inclusion of more functional traits. We argue, however, that the agreement of these three approaches provides support for consideration of niche overlap and for developing experimental collections and manipulations to test mechanistic explanations that would provide direct evidence of niche overlap.

Another potential limitation of our study is that a decade of data (2007–2017 for wood production and population dynamics, 2018–2019 for leaf production, and 2019 for fine root production) could be considered a short timeframe compared to the life of a tree and may potentially represent equal competitive effects and slow growth dynamics in young stands. While it is somewhat rare to experimentally manipulate tree species diversity in a common garden experiment specifically because of their long-life histories, the relative time scale here is not particularly different from previous work on more easily manipulated herbaceous plants or microbial communities. For example, it is routine to draw conclusions about niche partitioning from as little as one year of growth in experimental perennial grassland communities [18,30], even though perennial grassland plants may have lifespans comparable to many tree species [31]. Microbial communities are increasingly emerging as model systems for community ecology, and in those systems it is common to measure population dynamics on the scale of hours [32] while still seeking to gain insight into dynamics in nature that are more likely to play out much longer timeframes [33]. Since important insights and advances have been gained from other taxa on time scales that could also be considered short for the life history of those taxa, we argue that a decade of data on tree productivity and demography can, at the worst, provide as much insight as other previous short-term studies with different taxa.

### Conclusions

In conclusion, our results, combining experimental, observational, and statistical approaches in the study of coexistence, demonstrated that the three tree species used in this study, which occupy very similar niches within North American forests, potentially have overlapping niche spaces with implications for limited coexistence. The demonstrated effectiveness of combining multiple theoretical approaches may provide a framework for future analyses of species coexistence by exploring mechanistic tests of resource competition and fine-scale coexistence dynamics. The agreement or disagreement of these approaches can serve to generate hypotheses that future work should seek to falsify [34]. While the direct extension to broad ecological concepts from the outcomes of this work is limited due to the use of only three species in the current study, we suggest that if these independent methods for inferring coexistence continue to agree, community ecology might transition from a more descriptive science to a predictive approach. We recommend that more works seek to combine alternative approaches to continue to gain theoretical and predictive understanding of coexistence and community dynamics.

## 4. Materials and Methods

### 4.1. Study Species and Experimental Plot Design

We established an experimental planting of trees in west–central Indiana, USA (40°26′41.9″ N, 87°01′46.4″ W), in the spring of 2007. The main soil type is Rockfield silt loam, and the site is moderately well-drained. Mean annual temperature is 10.4 °C and mean annual precipitation is 970 mm [25]. The study species were three species of hardwood trees: *Prunus serotina* Ehrh. (black cherry), *Quercus rubra* L. (northern red oak), and *Castanea dentata* ((Marsh.) Borkh., American chestnut). Detailed life history characteristics for individual tree species can be found in Appendix A.

The focal species were combined in all seven possible combinations of one, two, or three species (Supporting Information Figure S2). The experiment also included three density treatments of 1 m, 2 m, or 3 m between trees which corresponded to 10,000 stems per hectare, 2500 stems per hectare, or 1111 stems per hectare, respectively, in a replacement series experimental design [25]. The seven diversity treatments and three density treatments were planted in a full factorial randomized split plot design where each replicate block contained three densities by seven combinations of species for a total of 21 plots per block. With three replicate blocks, this was a total of 63 plots (Supporting Information Figure S2). Complete details of the planting methods can be found in the Supplementary Information.

### 4.2. Productivity Estimates

Leaf production was estimated using litter traps to obtain leaf mass per area of ground in 2018 and 2019. Wood production was estimated using dendrochronology as the area of an annual ring known as a basal area increment (BAI). Cores were taken in 2018 and wood estimates were limited to 2007–2017. Root production was estimated using root in-growth cores in 2019. DP curves were fit using the lmer() from the lme4 package in R [35,36], and type III sums of squares were obtained from the lmerTest library where the denominator degrees of freedom were estimated using Satterthwaite’s method [37]. In all analyses, density was treated as a categorical factor, and species richness as a continuous covariate. Wood and leaf production also included year as a categorical variable. Random effects were individual trees nested in blocks for wood production, and litter trap stations nested in blocks for leaf production to control for repeated measures. A mixed model with a block as a random effect would not converge for root production, so we performed a simple generalized linear mixed effects model. Detailed descriptions of data collection and analysis are given in the Supplementary Information.

### 4.3. USDA Forest Inventory and Analysis Data

In 1999, the Farm Bill (PL 105-185) directed the US Forest Service to begin annual surveys of the national inventory of tree resources. These USDA FIA data are publicly available. They represent surveys from 124,563 permanent plots across the entire USA, allowing us to examine the co-occurrence of our three study species across almost their entire native range [38]. An FIA plot consists of four subplots with a radius of 7.3 m arranged in an equilateral triangle, with one subplot at the center, and the other subplots at the corners of the triangle with a total plot area of 669.7 m^2^. The identity and size of all trees with a diameter of ≥12.7 cm at a height of 1.37 m in each subplot were then recorded. An FIA plot of 670 m^2^ contains approximately 25 individual trees on average, and thus, we deemed this to be a reasonable scale to assess neighborhood-level species associations.

### 4.4. Checkerboard Score Analysis

The checkerboard score is named for the appearance of a perfect negative co-occurrence matrix, which takes on the appearance of a checkerboard based on the diagonal arrangement of 0 s and 1 s when species are perfectly segregated (i.e., $\left[\begin{array}{cc} 1 & 0\\ 0 & 1 \end{array}\right]$). To calculate the C-score, first, the number of checkerboard units for each pair of species is calculated as

(1) $$C_{ij}=\left(r_{i}-X_{ij}\right)\left(r_{j}-X_{ij}\right),$$

where $r_{i}$ is the number of times species i occurs without species *j*, $r_{j}$ is the number of times species j occurs without species i, and $X_{ij}$ is the number of sites where species i and j co-occur [20]. Typically, $C_{ij}$ is normalized by the total number of possible species pairs (P) and the total number of possible site pairs (N), which, using basic combinatorics, are given by

(2) $$P=\frac{S\left(S-1\right)}{2},$$

(3) $$N=\frac{n\left(n-1\right)}{2},$$

where *S* is the number of species, and *n* is the total number of replicate plots [20]. Across all sites, the normalized C-score is given by

(4) $$C_{n}=\sum_{j=0}^{S}\sum_{i<j}\frac{C_{ij}}{PN}.\;$$

To generate the null hypothesis that species randomly co-occur, we randomized the observed occurrence matrix from all FIA sites where our three focal species were present, and then repeated this randomization for a total of 1000 null matrices. The randomization procedure used a fixed–fixed randomization where row sums (species occurrences) and column sums (species occurrence within sites) were held constant because a fixed–fixed randomization has been shown to be more conservative and less prone to Type I errors [39,40]. Null community matrices were created using the permatswap function in the vegan library in R [41,42]. For each observed matrix, 1000 null matrices were sampled with 30,000 swaps between each sample and with a burn-in of 30,000 swaps prior to the first sample. We used the quasiswap method for generating null matrices, which does not produce sequential null matrices but instead generates a matrix at each time step that is fully independent of previous matrices [43]. The standardized effect size of the $C_{n}$-score can be calculated based on the observed $C_{n}$-score and the mean (*µ*) and standard deviation (σ) of the expected $C_{n}$-scores produced from the 1000 null matrices by calculating a z statistic [44] according to

(5) $$z=\frac{C_{n}-\mu }{\sigma }.$$

The z statistic is significant at the two-tailed p=0.05 level for −1.96<z>1.96.

### 4.5. Functional Traits and Soil Nutrient and Pollutant Analysis

Finally, to understand how the species differed in their functional ecology, we compared how species differed in height, leaf nitrogen, stem density, specific leaf area (i.e., leaf area per mass), seed mass, and total canopy leaf area. These six traits have been shown to explain 75% of variation in trait space among 46,085 globally distributed plant species [27]. We combined estimates of traits from our plots (Appendix A) with 1851 publicly available records from the TRY plant trait database [45]. We used principal component analysis (PCA) to identify similarities or differences among species in multivariate trait space [27,46]. The TRY database contains gaps in traits for all species, and gaps were species-specific; thus, to fill in gaps in trait data, we first took the mean trait value by species and by author from the combined trait data from TRY and our plots. We then imputed missing data using the imputePCA function from the missMDA package in R using the regularized method, and three components were used to predict the missing entries [47]. This library uses an iterative algorithm to estimate the missing PCA parameters and is robust to introducing error [47,48]. We used GLM to analyze differences in the PC scores of each species. Additionally, since our study only had three species, we also used PERMANOVA from the adonis2 function in the vegan library [41] to compare the species in the raw 6-dimensional multivariate trait space. Finally, we performed a detailed review of the natural history and morphology of the three species to better understand their similarities, differences, and role within a forest community (Appendix A) and assessed soil macro- and micronutrient and pollutant profiles using plant root simulator probes (Appendix A).

## Figures and Tables

**Table 1:** ANOVA table for the linear mixed models for leaf, wood, and root production. Note that root production was only collected in one year. Bold indicates significance at the α = 0.05 level.

Tissue	Treatment	F	df num	df den	*p*
Leaf	Diversity	1.50	1	120.8	0.2231
	Density	0.23	1	120.8	0.6325
	Year	2.75	1	83.2	0.1011
	Diversity × Density	0.17	1	120.8	0.6829
	Diversity × Year	0.53	1	83.2	0.4664
	Density × Year	0.05	1	83.2	0.8282
	Diversity × Density × Year	0.02	1	83.2	0.8870
Wood	Diversity	2.59	1	197.3	0.1094
	Density	0.45	1	197.7	0.5012
	**Year**	**35.40**	**10**	**1733.0**	**<0.0001**
	Diversity × Density	2.41	1	197.3	0.1220
	Diversity × Year	0.41	10	1732.9	0.9434
	Density × Year	0.79	10	1733.0	0.6348
	Diversity × Density × Year	0.71	10	1733.0	0.7136
Root	Diversity	0.17	1	56	0.6787
	Density	<0.01	1	56	0.9745
	Diversity × Density	0.13	1	56	0.7193

## Data Availability

The USDA FIA data are publicly available from the USDA, and the TRY data are also publicly available. Our site-specific productivity and demography data and code used for analyses are available on request from the corresponding author.

## Supplementary Materials

The following supporting information can be downloaded at: https://www.mdpi.com/article/10.3390/plants14152271/s1. Table S1: Regression model for species diversity versus leaf production shown in Figure 1A,B; Table S2: Regression model for species diversity versus wood productivity shown in Figure 1C,D; Table S3: Regression model for species diversity versus root productivity data shown in Figure 1E,F; Table S4: ANOVA comparisons of PC1 and PC2 of trait data analyzed by PCA and PerMANOVA outcome; Table S5: ANOVA analysis outcomes of soil nutrient and pollutant profiles for the experimental planting; Figure S1: Species trait differences in ordination space; Figure S2: Plot layout of study site; Figure S3: Pairwise scatter plots (lower triangle), correlation matrix (upper triangle), and density distributions (diagonal) of the imputed and transformed trait data that was used for the PCA of species trait differences.

## Appendix A

Additional method descriptions of study design and collections and detailed life history of study species.

### Appendix A.1. Details of Planting Methods

In 2007, trees were planted as bare-root seedlings. Seedlings were obtained from Cascade Forest Nursery in Cascade, Iowa, USA. The *Q. rubra* and *P. serotina* nursery seed source was from stands local to the nursery. Pure *C. dentata* seeds were collected from a forest stand near Galesville, Wisconsin, USA [25]. Prior to planting, 2% glyphosate was applied to eliminate herbaceous vegetation in the plots. After planting, a 3 m high fence was erected around the perimeter of the entire experiment to prevent damage from deer. The fence was removed in 2012 when trees were tall enough to avoid deer browsing on twigs and leaves. From 2008 to 2010, a mixture of pendimethalin and glyphosate was applied in the rows between trees with a backpack and side-mounted band sprayer as needed to reduce competition from herbaceous weeds as the seedlings established. In 2011, instead of spraying herbicide, the rows were mowed. Starting in 2012, weed control was discontinued because the trees were taller than herbaceous weeds.

Each individual plot always contained 56 trees arranged in a 7-by-8 grid (Figure S2). Thus, plot dimensions depended on the density treatment. Plots planted with trees at a 1 m spacing were 7 by 8 m (54 m^2^), plots planted with trees at 2 m spacing were 14 by 16 m (224 m^2^), and plots planted with trees at 3 m spacing were 21 by 24 m (504 m^2^). In total, the 63 plots take up approximately 2.4 ha. In the mixed-species plots, trees were planted sequentially across the row with seven trees in a torus wrap such that individuals of the same species were never planted next to each other on the grid (e.g., Figure S1B). The 26 perimeter trees around each plot were denoted as a buffer row to mitigate potential edge effects and were never measured, leaving 30 focal trees in the middle of each plot (Figure S1B). Across the entire experiment, there were 1648 buffer trees and 1800 focal trees for a total of 3438 trees. All of the 1800 focal trees were individually tagged and numbered.

As of 2018, the stand was a closed canopy forest with trees approaching 10 m in height, up to 20 cm in diameter at breast height, and all trees were at reproductive maturity. Chestnut blight (*Cryphonectria parasitica* (Murrill)) was detected in the plots for the first time in 2017, and thus our estimates of wood productivity and population trajectories were limited to 2007–2017 to focus on ecological interactions among the tree species without the influence of chestnut blight. Only the estimates of root and leaf production and some trait estimation occurred after 2017. By 2023, nearly all *C. dentata* had died or were infected with blight.

### Appendix A.2. Detailed Data Collection Methods

Wood production between 2007 and 2017 was estimated using dendrochronology on basal cores taken during the 2018 growing season. Of the 1800 total focal trees in the experiment, three of each species in each plot were randomly selected to be sampled for a total of 24 cores per species and 72 cores in total. A flooding event in the southern-most 3 m spaced plots led to significant mortality of trees (Figure S1C), and thus the 3 m plots were dropped from most productivity analyses due to lack of replication. Cores were air-dried in the lab, mounted on wood blocks, and sanded flat with sequentially finer sand-paper ending with 800 grit. Cores were imaged at a resolution of 1200 dots per inch. Ring widths on digitized images were measured in CooRecorder (v 9.00, Cybis Elektronik & Data AB, Saltsjöbaden, Sweden). Assuming the cross-sectional area of the trunk was a circle, we estimated basal area in each year. The area of a ring grown in year t was therefore estimated as the basal area at the end of year t, minus the basal area at the end of year t−1, creating a basal area increment (BAI) in each year as an estimate of wood production. BAI was log-transformed to achieve normality. The data analysis included the fully factorial combination of year by species richness by density as fixed effects in a linear model where year and density were treated as factors, but species richness was treated as a continuous covariate. Individual tree number and block were combined as a random effect to control for repeated measures. The linear model was fit using the lmer() from the lme4 package in R [35,36], and type III sums of squares were obtained from the lmerTest library where the denominator degrees of freedom were estimated using Satterthwaite’s method [37].

Starting in 2018, leaf litter was collected in litter traps, which were 28.6 cm in diameter, 36 cm deep, and had eight 1.3 cm holes drilled into the bottom for drainage. In 2018 and 2019, leaf litter collection began in September, and continued bi-weekly until December. Upon return to the lab, leaves were sorted by species, dried, and weighed. Plot size differed by tree density, and so we applied sampling effort proportional to the plot size. This resulted in a grid of leaf litter traps spaced 6 m apart across the site with one litter trap in the center of the 1m spaced plots, four in the center of the 2 m spaced plots, and nine in the center of the 3 m plots. In 2018 we had a total of 258 sampling stations. Since sampling occurred every two weeks across three months, this generated 1548 samples that were sorted by species. Using the 2018 data, we determined that the number of litter traps could be reduced to one leaf litter sampling station in the 1m spaced plots, two in the 2 m spaced plots, and three in the 3 m spaced plots with no change in estimated mean or variance at the plot level. Thus, in subsequent years, we randomly removed some sampling stations, and in 2019, only 114 leaf litter stations were sampled across three months in the autumn. Total untransformed leaf production in each year was analyzed by summing across the six sampling dates, and all species within each plot, divided by the area of the litter traps (0.064 m^2^). This calculation provided an estimate of leaf production per square meter per year. The data analysis was the same as above for wood, except litter trap station and block were combined as a random effect to control for repeated measures.

Fine root production in 2019 was estimated using root in-growth cores. In this method, a 7.5 cm diameter and 50 cm deep cylindrical soil core was extracted from the ground in the autumn of 2018. The living roots were sorted out of the soil and then the root-free soil was replaced inside a size 7 mesh cylinder. The in-growth cores were paired with litter trap stations, and so there was 1 in each 1 m plot, 2 in each 2 m spaced plot, and 3 in each 3 m spaced plot for a total of 114 in-growth cores. After one year, in the autumn of 2019, we retrieved the in-growth cores. These were sorted into the top 10 cm, the middle 20 cm, and the bottom 20 cm, dried and weighed. Analyses summed all three depths within a plot and divided by the total surface area of the soil at the top of the core (0.0044 m^2^) to obtain an estimate of fine root production per year per square meter. Root production was log-transformed to achieve normality. The data analysis included density as a factor, and species richness as a continuous variable (only one year of root data was measured). For some reason, the mixed effects model would not converge with block as a random effect. Since there were no repeated measures within a plot for the roots (one sample per plot), we dropped block from this model to analyze only a fixed effects model. A type III sum of squares was obtained using the Anova() function from the car library in R [49].

### Appendix A.3. Measurement of Functional Traits in Our Plots

Total leaf area was estimated from the litter traps in monocultures only. We took the total leaf mass per the area of the trap opening, scaled it up to the size of each plot, divided by the number of trees in a plot, and then multiplied by the mean SLA of each species in the TRY data to estimate total canopy leaf area per tree. Only one value per plot and year was estimated. The total leaf area values downloaded from TRY were from saplings, not mature reproductive trees, and these were not used.

Tree height in our plots was estimated using a terrestrial LiDAR scan (Leica BLK360, Lecia Geosystems, Heerbrugg, Switzerland). A single scan was taken in the center of each plot, and the point cloud was cropped to eliminate points outside the boundaries of the plot borders. Scans occurred between 21 July and 20 August 2020. Height of each species was estimated only from monocultures and we used the single maximum LiDAR return detected as height. This gave us nine height estimates for each species, one from each monoculture.

Foliar nitrogen (N, % dry mass) and specific leaf area (SLA; cm^2^ g^−1^) were generated from leaf-level hyperspectral data using fresh-leaf spectroscopic models from [50]. In 2018 and 2019, foliar samples from all tree species across all plots available were collected in June, August, and October using pole pruners. In each plot, between seven and twenty leaves, depending on leaf size, were sampled from each tree species from the upper and lower canopies of each tree. Hyperspectral data were collected on leaves using a full-range spectroradiometer (SVC 1024i, Spectra Vista Corporation, Poughkeepsie, NY, USA) on three separate leaf sections per leaf and averaged to one spectral measurement per monoculture plot for PCA.

The TRY data was used for specific leaf area (SLA), not leaf mass per area (LMA). It is common for many authors to write that SLA=1/LMA (this was also written in the TRY data file), but this is not true when either value represents a mean. Importantly, the mean of a set of ratios is not equivalent to the mean of the inverse of that same set of fractions (e.g., $\frac{\frac{1}{4}+\frac{1}{2}+\frac{3}{4}}{3}=\frac{1}{2}$, while $\frac{\frac{4}{1}+\frac{2}{1}+\frac{4}{3}}{3}=\frac{22}{9}$, and 0.5 is not equal to $\frac{9}{22}$; this is true for any mean of ratios). Thus, we did not convert SLA to LMA as has become more common and is likely a very large source of error among studies that make this basic mathematical mistake.

Despite our best efforts, we could not seem to get to seeds before foraging animals to estimate seed mass. Also, we were concerned about chestnut blight affecting estimates of stem density by the time we were ready to estimate traits. Thus, we did not estimate seed mass or stem density from our plots, and rely on TRY for these trait data. These are likely the least variable traits from site to site.

Soil macro- and micronutrient profiles and common pollutants were assessed in 2018 using plant root simulator probes (PRS; Western Ag, Saskatoon, SK, Canada). Nitrate, ammonium, nitrogen, calcium, magnesium, potassium, iron, manganese, copper, zinc, boron, sulfur, lead, aluminum, and cadmium were measured and the number and placement of PRS probes was the same as the leaf litter collection buckets described above. No macro- and micronutrient or pollutant models were statistically significant (Table S5).

### Appendix A.4. Detailed Description of Study Species

#### Appendix A.4.1. *Quercus rubra* (L.)

*Q. rubra*, the northern red oak, is a late successional species in the Fagaceae family. Its distribution ranges from Nova Scotia to Ontario, Canada, in the north, and in the south from Nebraska down through Oklahoma and east to North Carolina, USA [51,52]. *Q. rubra* can survive in a variety of soil, from clay to loamy sands and some have a high content of rock fragments, such as soils derived from glacial material, residual sandstones, shale, limestone, gneisses, schists, and granites [51,52]. Northern red oak generally prefers well-drained acidic soil with a thick A horizon and a loam-to-silt loam texture [53,54]. Further, they tend to grow on concave slopes, deep ravines, and well-drained valley floors [51,53].

*Q. rubra* has polycyclic growth, meaning it can have multiple leaf flushes in a growing season. In addition, they are known to grow rapidly, averaging about 61 cm in height annually for ten to thirty years [51,55]. At their mature height, *Q. rubra* are between 20 and 30 m tall with a large DBH of 61 to 91 cm when left in undisturbed forests . The crown uniformity is symmetrical with a round dense shape [53]. When grown in dense forest, the trees tend to develop a tall, straight columnar bole and large crowns, while open-grown trees tend to have short boles and spreading crowns [53]. The trees also prefer full sun but do have some limited shade tolerance [51,53,54]. Notably, it is also drought-tolerant with a high capacity to tolerate desiccation, despite high stomatal density [53]. Further, the trees are tolerant to low temperatures, down to negative 40 °C in some cases [53].

This species rapidly resprouts when cut, with almost 95 percent of the northern red oaks in new production stands being sprouts from advanced reproduction or from stumps of cut trees [51,53,54]. Large stumps tend to produce more sprouts than small ones but by about age twenty to twenty-five, the number of living sprouts per stump averages four or five regardless of parent tree or stump size [51,53,54]. However, for seeds, seeding is generally irregular, about every 2 to 5 years after 25 years, while some trees can produce as many as 500 or more acorns with only about 1 percent germinating due to 80 percent of acorns being eaten by various animals [51,52,53,54,55,56]. The fruits are the acorns themselves, and are partially enclosed by a scaly cup, they are round or ovular at 2.54 to 12.7 cm in length, and the seeds are also hard and dry, produced from brown wind pollinated catkins [56]. The acorns are hypogeal; they turn brown when mature and ripen after two years from late August to late October [51,57]. *Q. rubra* is monoecious. Male flowers grow in groups of three 5 to 10 cm long green catkins with multiple stamens and a calyx concealed by hairy bractlets [51,52,53,55,56,57]. Female flowers are ovoid and consist of a single ovary with multiple stigmas. They are produced in clusters varying from one to five [56]. Its staminate flowers grow from catkins that develop from the leaf axils of the previous year [51,53,54,55,56]. The catkin emerges before or at the same time as the current leaves, expanding in April or May [51,54,56]. The pistillate flowers can be solitary or grow in multiple flowered spikes that develop in the axils of the current year’s leaves [51,57].

Leaves are deciduous and simple with pinnate ventilation; they maintain an elliptic–oblong shape that can be 10 to 20 cm long and 8 to 15 cm wide with 7 to 11 waxy sharp pointed leaves that maintain bristly tips [53,55,57]. The leaves are known for their showy colors, going from green in the spring to red in the fall and brown in the winter [51,53,54,57].

*Q. rubra* maintains a strong, deep taproot that develops when the tree is young and can reach over 70 cm deep at three to five years of age; they also grow thin shallow lateral roots that can expand horizontally to 15 m around the tree stem, with root growth that is continuous throughout the vegetation season [51,54,57]. Notably, when in rich sandy soils, with a shallow water table, it does not develop a taproot but instead develops lateral roots with many oblique or vertical branches, and in sandy soils overlying a loam layer, the trees develop a deep and wide root system [54].

#### Appendix A.4.2. *Prunus serotina* (Ehrh.)

*P. serotina*, the black cherry, is an early successional, gap-phase species in the Rosaceae family. Its range includes most of the Midwest through the eastern half of North America, including Nova Scotia Canada in the north down to Southwest Florida in the south. *P. serotina* is an aggressive colonizer that prefers soils that are strongly acidic, infertile, and have a coarse fragment content [58].

*P. serotina* is a deciduous, single-stemmed tree, often between 28 and 38 m in height [58,59]. Black cherry is a generally shade-intolerant species that primarily occurs as scattered individuals in various types of mesic woods and second-growth hardwood forests, successional vegetation, and forest openings, and in old fields and along fencerows [58,59,60]. Despite being considered shade-intolerant, it can still persist under the canopy of other larger trees, but the induced shading generally does not allow the tree to fruit. In forests they grow best within codominant crown classes, or in sun-filled canopy openings [58,59,60]. The tree crown is often an oval shape of irregular uniformity and a moderate density, with branches that lay so low they often touch the ground; its canopy can also stretch wide, reaching 9 to 15 m [57,58,59,60]. Notably, the leaves, twigs, bark, and seeds produce a cyanogenic glycoside, limiting herbivory and competition [61].

*P. serotina* have a high regeneration rate from stumps, growing and dying back as needed until adequate sunlight is available, allowing the tree to survive for up to one hundred years [58]. It is common for seedlings and saplings to develop specifically under openings made by the death of a mature tree [58,59]. Seeds are oblong ovid stones around 0.75 cm long; they are dispersed from August to September produced on trees at about 10 years of age but produce the most seeds after 30 years; seeds can be produced yearly but usually at one-to-five-year intervals [58,59]. Seeds require a winter of stratification to occur before germination, but germination rates are high, growing at 5 to 10 cm only thirty days after germination, allowing the trees to dominate post climax communities [58,59].

Leaves are leathery in texture, alternate and simple with pinnate-owed leaf venation, a serrate leaf margin, and an oblong–ovate shape at about 5 to 15 cm long and 2.5 to 5 cm wide; they have finely toothed margins that are glabrous and can often have red hairs along the midrib near the base [57,58,59]. The leaf generally has one or two glands on the petiole near the leaf blade with buds that are 0.3 to 0.6 cm long [57,58,59].

Black cherry is monecious and insect-pollinated. The inflorescence is an oblong–cylindric raceme of 10 to 15 cm in length that sits at the end of leafy twigs that produce numerous flowers [59]. Flowers are white, 8 to 10 cm long, with a calyx tube of short lobes and five petals and can be in groups of 30 flowers at the end of each raceme [58,59]. Flowering occurs late in relation to leaf development, around mid-May after leaves are fully developed, hence the name “serotina”, meaning late-appearing [57,58,59].

Fruits are globular red, purple, or black drupes of about 1.2 cm in diameter and produce one ovoid stone seed 6 to 8 mm long [57,58,59,60]. The fruits are considered important resources to a variety of animals which consume the fruit, spreading the seed long distances and aiding in germination [57,58,59,60]. Seedlings develop a taproot that reaches 15 to 20 cm into the soil, with multiple lateral roots [57,58,59,60]. Taproots only persist during early stages of development, eventually giving way to a vast-spreading but shallow root system that spreads around the upper 61 cm of the surface of the soil, but can rarely go as deep as 120 cm [57,58,59,60]. Notably, the roots of *P. serotina*, like its shoots, have cyanogenic glycoside in order to persist against pests and other plant competition [61].

#### Appendix A.4.3. *Castanea dentata* ((Marsh.) Borkh.)

*C. dentata*, the American chestnut, belongs to the Fagaceae family. It was once the dominant riparian species in North American deciduous forest, covering 200 million acres, comprising 25 to 50% of the forest canopy [62,63,64]. As such, it was probably a late successional species. Its historic range reached from southern Maine in the USA to Michigan in the east, and south to Alabama and Mississippi. It is a drought-tolerant species and can be found in almost any soil type. However, it grows best in well-drained, subxeric-to-mesic soils often found in sand plains and dry ridges. Additionally, it grows poorly in very wet or very dry soils, or areas with high pH or on limestone-derived soils [64]. It tolerates nutrient-deficient and fertile soils, with trees showing improved growth with increasing nutrient level [65]. While able to survive in pure stands, it is a dominant species in almost any community it grows in and is also commonly found in combination with a variety of other species [65].

*C. dentata* is a tall and fast-growing species and can grow between 10 and 25 mm per year [62,63,64,65,66]. Mature trees can reach 18 to 27 m tall and 91 to 152 cm in diameter, though individuals as large as 213 cm in diameter and 37 m tall have been recorded. A sun-loving, broadleaf canopy species, the branches are wide-spreading with a rounded crown that helps it shade out competitors; it can reach its maximum height after only twenty years [63,64]. Additionally, saplings can sprout from the root collar and grow rapidly with an average height of 3.7 m at five years of age. Saplings are somewhat shade-tolerant, and exhibit plasticity by increasing leaf mass per area with greater light availability; light availability is shown to have higher impacts on growth than soil parameters [63,64,65]. Sprouts can lay dormant for several years before development and can sprout from trees that are up to one hundred and seventy years old; their ability to die back and regrow is critical for survival against a lack of sunlight [63,65].

Leaves are alternate, simple, and can be oblong-to-lance-shaped and coarsely serrated; the bristle-tipped teeth tend to point forward sharply; they are about 5 cm wide and 13 to 20 cm long with a short and stout petiole that is enlarged at the base [66]. Notably, the leaves have been found to be allelopathic, with phytotoxic chemicals sufficient to inhibit the germination of co-occurring species [63].

*C. dentata* is a monoecious, self-incompatible species that generally flowers from May to July after the last frost and continues after leaf out. Flowers are borne on ascending spike-like aments that are either staminate or bisexual; the staminate aments are about 13 cm long, with flowers in clusters of three to seven along the ament axis [63,65]. Shoot flowers are spatially separated such that lower lateral shoots will consist of all male flowers, but the stronger upper vertical shoots have male flowers on upper shoots and female flowers on lower shoots [63,65]. Previous natural history observations suggested that male inflorescences had characteristics of an insect pollinated flower, but current research shows that wind pollination is the primary mechanism of pollen dispersal [63]. Pollen is released at different times with the unisexual inflorescences releasing pollen before female receptivity and the bisexual inflorescences after receptivity; however, being self-incompatible and having a short distance of pollen dissemination means that they must be at a maximum distance of about 100 m from each other for successful pollination [63,65].

The fruit matures between September and October and is produced as one to three nuts 3 cm in diameter and 2 cm long, encapsulated in a spiny involucre that is usually 6 cm in diameter. A mature tree can produce around six thousand nuts per year [63,65,66]. However, as seeds are extremely important to wildlife, most seeds are consumed, leaving only one to five seeds to germinate into seedlings per reproductive adults. Notably, in greenhouse experiments, there is a ninety percent germination rate [65].

There is little and contradicting information on the root systems of American chestnut. Some resources indicate a deep taproot while others indicate a shallow lateral root system, with lateral roots being reported as deep as 1 m into the soil, and large taproots being reported as reaching 1 m to 2 m deep into the soil [65]. Regardless of root type, the roots are susceptible to a fungal disease if the soil is too moist, which is why *Castanea dentata* generally prefers dry soil [63,65]

The invasive fungal chestnut blight pathogen (*Cryphonectria parasitica* (Murrill)) was accidentally introduced to North America in 1904 [63]. Mortality rates to this novel enemy were nearly 100% and this once competitive forest species is now critically endangered. Notably, the trees are less susceptible to blight when young, but when reaching a height of 15 m with a DBH of 20 cm they are susceptible to infection from wounds or cracks in the bark [67]. Infection began at our site in 2017.
