# Supplementary material for: Niche Overlap in Forest Tree Species Precludes a Positive Diversity–Productivity Relationship
Source: Plants (Basel). 2025 Jul 23;14(15):2271. doi: 10.3390/plants14152271 (PMC12348305; doi:10.3390/plants14152271)
Supplement: Supplementary file 1 [file plants-14-02271-s001.zip › plants-3554043-supplementary.pdf]

SUPPLEMENTARY TABLES AND FIGURES

Table S1: Regression model for species diversity versus leaf production shown in Figure 1A,B. The model was lm(Leaf.production~ density * diversity * year) in R. The overall regression was not significant ($F_{7,160} = 1.66$, p=0.1217), and critically, none of the individual slopes for the diversity productivity relationship were significantly different from zero.

Coefficients	Estimate	SE	t	p
Intercept	260.6	51.2	5.1	<0.001
Diversity	21.3	27.7	0.77	0.443
Density(2m)	-10.5	57.3	-0.18	0.855
Year(2019)	76.2	72.4	1.0	0.295
Diversity*Density(2m)	6.0	30.9	0.19	0.847
Diversity*Year(2019)	-19.8	39.1	-0.51	0.614
Density(2m)*Year(2019)	-13.0	84.9	-0.15	0.88
Diversity*Density(2m)*Year(2019)	2.71	45.9	0.06	0.953

Table S2: Regression model for species diversity versus wood productivity shown in Figure 1C,D. The model was lm(BAI ~ density * diversity * year) in R. The overall regression was significant ($F_{43,1925}$ = 24.1, p<0.0001), though the data contained a large amount of unexplained error (R^2 = 0.33). None of the individual slopes for the diversity productivity relationship were significantly different from zero.

**Coefficients**	**Estimate**	**SE**	**t**	**p**
Intercept	0.58	2.05	0.284	0.7761
Density(2m)	0.39	2.50	0.154	0.8773
Year(2008)	0.08	2.33	0.034	0.9728
Year(2009)	0.93	2.29	0.405	0.6859
Year(2010)	1.10	2.28	0.484	0.6283
Year(2011)	1.81	2.27	0.799	0.4246
Year(2012)	2.57	2.26	1.134	0.2567
Year(2013)	2.60	2.26	1.147	0.2516
Year(2014)	4.05	2.26	1.788	0.074
Year(2015)	4.29	2.26	1.892	0.0586
Year(2016)	5.24	2.26	2.314	0.0208
Year(2017)	4.79	2.26	2.114	0.0346
Diversity	-0.08	0.94	-0.087	0.9307
Density(2m):Year(2008)	0.28	2.96	0.094	0.925
Density(2m):Year(2009)	0.25	2.88	0.086	0.9312
Density(2m):Year(2010)	0.66	2.87	0.229	0.8193
Density(2m):Year(2011)	1.52	2.86	0.533	0.5943
Density(2m):Year(2012)	2.51	2.85	0.88	0.3787
Density(2m):Year(2013)	1.68	2.85	0.592	0.5541
Density(2m):Year(2014)	1.27	2.85	0.447	0.6546
Density(2m):Year(2015)	2.61	2.85	0.918	0.3585
Density(2m):Year(2016)	1.46	2.85	0.514	0.6076
Density(2m):Year(2017)	2.14	2.85	0.75	0.4531
Diversity:Density(2m)	-0.08	1.16	-0.066	0.9472
Diversity:Year(2008)	-0.01	1.07	-0.009	0.9931
Diversity:Year(2009)	-0.17	1.05	-0.162	0.8712
Diversity:Year(2010)	-0.06	1.05	-0.057	0.9542
Diversity:Year(2011)	-0.25	1.04	-0.239	0.8108
Diversity:Year(2012)	-0.15	1.04	-0.143	0.8866
Diversity:Year(2013)	0.03	1.04	0.026	0.9791
Diversity:Year(2014)	-0.46	1.04	-0.443	0.6576
Diversity:Year(2015)	-0.40	1.04	-0.38	0.7037
Diversity:Year(2016)	-0.69	1.04	-0.659	0.51
Diversity:Year(2017)	-0.26	1.04	-0.25	0.8025
Diversity:Density(2m):Year(2008)	0.02	1.38	0.011	0.9913
Diversity:Density(2m):Year(2009)	0.07	1.34	0.052	0.9587
Diversity:Density(2m):Year(2010)	0.12	1.34	0.09	0.9284
Diversity:Density(2m):Year(2011)	-0.04	1.33	-0.034	0.9727
Diversity:Density(2m):Year(2012)	0.39	1.33	-0.295	0.7681
Diversity:Density(2m):Year(2013)	0.25	1.33	0.186	0.8523
Diversity:Density(2m):Year(2014)	0.81	1.33	0.614	0.5396
Diversity:Density(2m):Year(2015)	0.26	1.33	0.197	0.8438
Diversity:Density(2m):Year(2016)	0.80	1.33	0.606	0.5446
Diversity:Density(2m):Year(2017)	0.82	1.33	0.619	0.5358

Table S3: Regression model for species diversity versus root productivity data shown in Figure 1E,F. The model was lm(Root.production~ density * diversity) in R. The overall regression was not significant ($F_{3,56} = 0.27$, p=0.8476), and critically, none of the individual slopes for the diversity productivity relationship were significantly different from zero.

**Coefficients**	**Estimate**	**SE**	**t**	**p**
Intercept	121.5	28.5	4.3	<0.0001
Diversity	-8.3	15.1	-0.55	0.585
Density(2m)	-2.8	34.9	-0.08	0.936
Diversity*Density(2m)	6.7	18.5	0.364	0.717

Table S4: Upper rows: ANOVA tables for the linear models fit to trait data to compare PC scores from the first two axes among species. Lower row: PerMANOVA on imputed raw trait value differences among species.

Trait	F	Num df	Den df	p
PC1	2.71	2	104	0.0709
PC2	2.11	2	104	0.1263
PerMANOVA	1.74	2	104	0.1724

Table S5: ANOVA comparison of individual soil nutrient profiles collected from 259 sampling points, averaged to one value per plot, in the experimental planting in 2018. None of the models were statistically significant so no individual factors (species richness, planting density, or there interaction) were considered.

**Compound**	**model *p*-value**
Nitrate	0.557
Ammonium	0.847
Nitrogen	0.496
Calcium	0.068
Magnesium	0.208
Potassium	0.644
Phosphorous	0.542
Iron	0.428
Manganese	0.628
Copper	0.575
Zinc	0.742
Boron	0.910
Sulfer	0.711
Lead	0.587
Aluminum	0.560
Cadmium	0.782

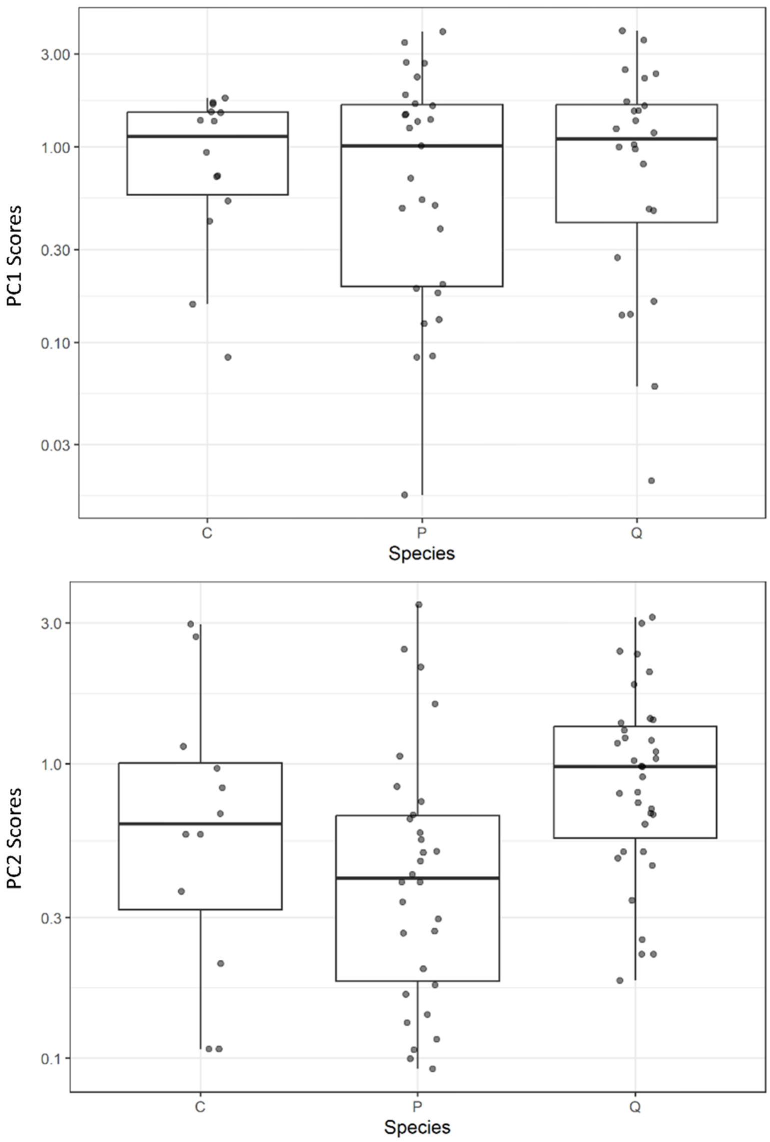

Figure S1: Species trait differences in ordination space. Comparisons of species trait differences using a combination of publicly available TRY data, and traits measured in our plots. None of these differences were statistically significant when analyzed using multivariate approaches (PCA or PERMANOVA; Supporting Information Table S4)

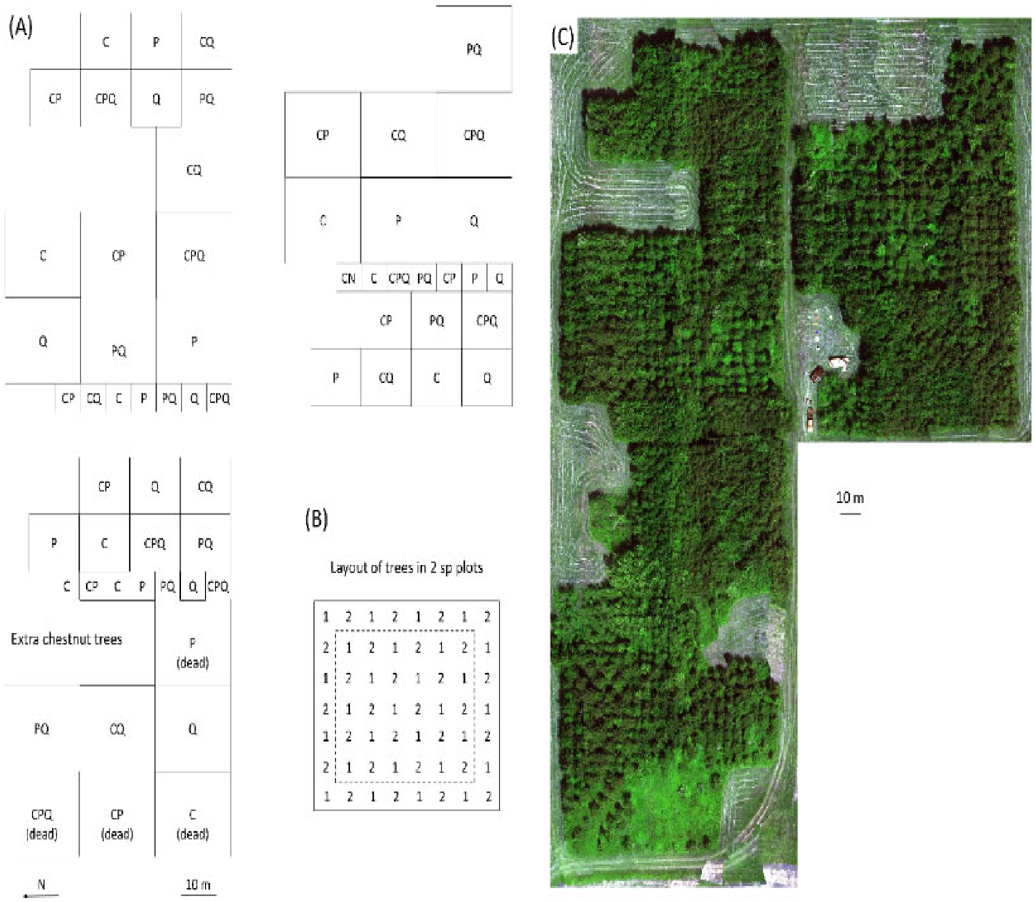

Figure S2: Plot layout of study site. **(A)** The experiment has three replicate blocks that include all possible combinations of *C. dentata* (C, American chestnut), *P. serotina* (P, black cherry) or *Q. rubra* (Q, northern red oak), at three different planting densities (1m, 2m or 3m between trees; 1 tree/m^2, 0.5 trees/m^2, or 0.33 trees/m^2 respectively) in a full factorial split plot design. Unfortunately, many trees in the southern-most 3m spacing plots died due to an unusual flooding event. These plots are not included in productivity data due to inadequate sample size for analysis, however these plots are included in demography data. Surplus chestnut trees were planted in an open space at the southern end of the experiment because of their conservation value. **(B)** Each plot contains 56 trees that alternate between species (indicated by 1 and 2). There is a buffer row of trees that are not measured around each plot (dashed line) leaving 30 focal trees in the center of each plot, and 1260 individual focal trees in 1m and 2m spaced plots. (C) An aerial image of our plots showing the basic layout. The dead 3m spaced plots, and the extra chestnut trees that are not part of the experiment, are especially evident in this image. Field vehicles are also visible in the center of the image.

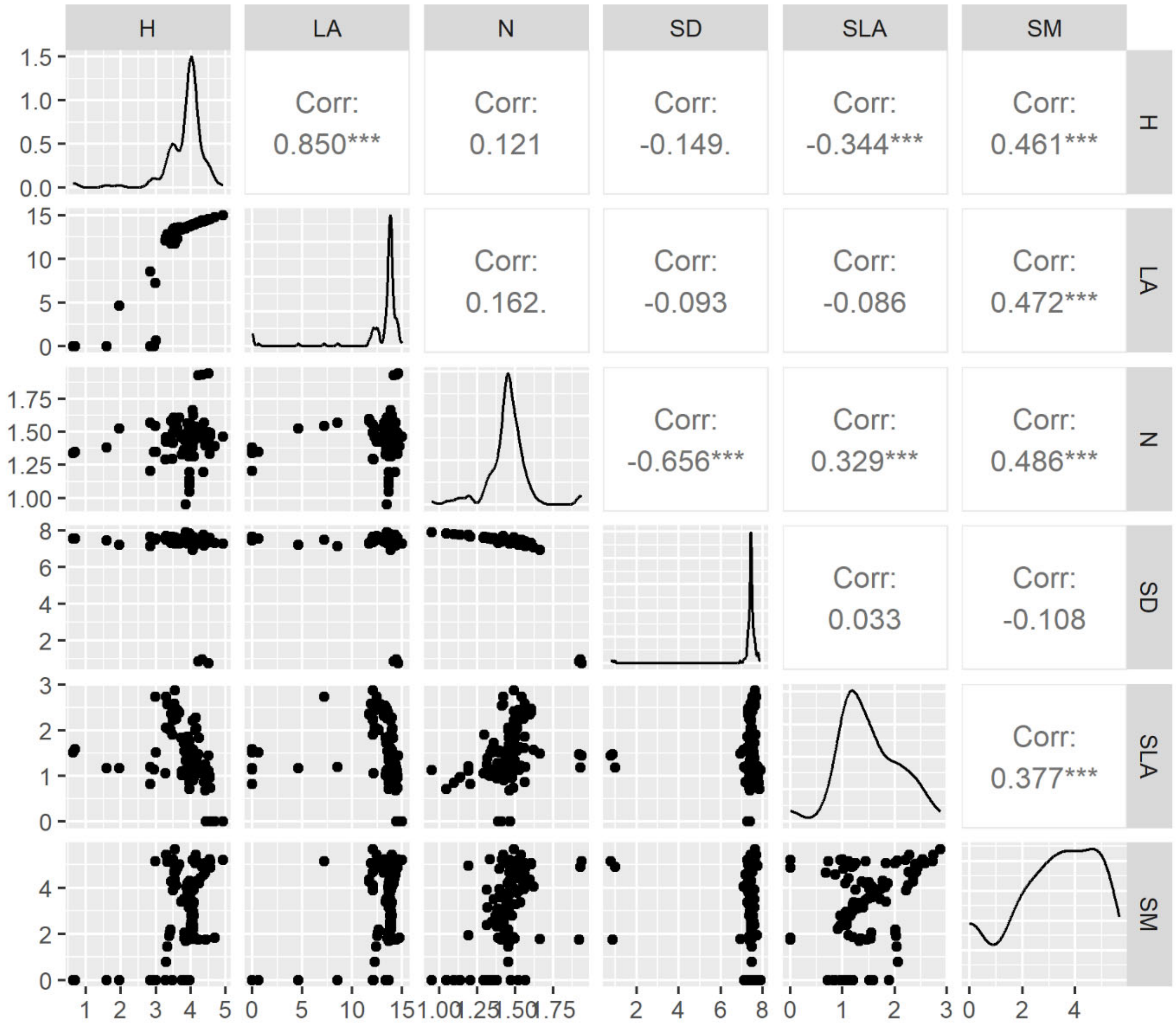

Figure S3: Pairwise scatter plots (lower triangle), correlation matrix (upper triangle, and density distributions (diagonal) of the imputed and transformed trait data that was used for the PCA analysis of species trait differences.
